# Factors affecting utilization of cervical cancer screening services among women attending public hospitals in Tigray region, Ethiopia, 2018; Case control study

**DOI:** 10.1371/journal.pone.0213546

**Published:** 2019-03-14

**Authors:** Hirut Teame, Lemlem Gebremariam, Tsega Kahsay, Kidanemaryam Berhe, Gdiom Gebreheat, Gebrehiwot Gebremariam

**Affiliations:** 1 Department of Public Health, College of Medicine and Health Science, Adigrat University, Adigrat, Ethiopia; 2 Department of Medical Laboratory Technology, College of Medicine and Health Science, Adigrat University, Adigrat, Ethiopia; 3 Department of Nursing, College of Medicine and Health Science, Adigrat University, Adigrat, Ethiopia; Ordu University, TURKEY

## Abstract

**Background:**

Incidence and mortality of cervical cancer is the leading cancer among women in Ethiopia. Absence of effective detection methods and treatment strategies is a major reason for the sharply rising cervical cancer rates in developing countries.

**Objective:**

To determine factors affecting utilization of cervical cancer screening services among women attending public hospitals in Tigray region in 2018.

**Methods:**

Hospital based unmatched case control study was applied with sample size of 312 cases and 312 controls. Data was entered to Epi data version 3.1 and exported to SPSS version 20. The odds ratio with their 95% confidence interval, two-tailed P value was calculated. Variables with P value ≤ 0.05 in the bivariate analysis were included in the multivariate logistic regression model.

**Results:**

Being in the age group of 30–39 and 40–49 years were two and four times more likely to utilize cervical cancer screening than those who were 21–29 years (AOR = 2.15 95%CI:1.11, 4.17 and AOR = 3.86 95%CI:1.48, 10.06) respectively. Current occupation with governmental and private employee were four and three times more likely to utilize the screening service than those housewife respectively (AOR = 3.85 95%CI: 1.87, 7.92 and AOR = 3.17 95%CI: 1.31, 7.66). Having ever given birth and history of multiple sexual partners were more likely to utilize the screening service (AOR = 2.57 95%CI: 1.02, 6.50) and (AOR = 2.65 95%CI: 1.10, 6.40) respectively.

**Conclusions:**

There is a need to strengthen policy and guidelines on cervical cancer screening among women particularly with regarding age group of 21–29, current occupation with housewife, single sexual partner and null parity. All stakeholders should give priority on the promotion and initiation of women to acquired good knowledge and attitude on cervical cancer screening.

## Introduction

The cervix is the lower part of the uterus, the place where a baby develops during pregnancy. The cervix can be affected by different diseases and cancer is one of it [1]. Cervical cancer is the abnormal growth of cells that have the ability to invade other part of the body [2]. The global disease burden of cervical cancer is estimated at half a million new cases and 230000 deaths every year and nearly 79000 new cases occur in Africa each year [3]. It is the second most common cancer among women in the developing countries [4]. The leading cause of cancer mortality and approximately 7100 are diagnosed each year among Ethiopian women [5]. Cervical cancer screening is looking for precursors, before a person has any symptom and if abnormal tissues or cancer is found early, it may be easier to treat or cure [6]. Evidence indicates that significant portion of the burden of cervical cancer is potentially prevented by early screening and treatment [7–9]. However, there are various factors that affect the utilization of cervical cancer screening services by women [7–10]. In the case of Ethiopia cervical cancer screening practices is low, study among female health care providers shows 17% of them ever screened for cervical cancer [11]. Study among reproductive health service clients in Addis Ababa shows 3.5% of women were screened for cervical cancer in their life time [12]. Study in Mekelle shows that only 10.7% nurses reported that they have ever been tested for cervical cancer in five years duration (2009–2014) [13]. However, the factors affecting utilization of cervical cancer screening services are not well identified in our setup.

Knowing factors affecting utilization of cervical cancer screening services helps to take an action in each factor to decrease the morbidity and mortality of cervical cancer. And allocate resources, for decision at different level so as to control the problem and concurrent losses coming with the disease burden. Hence, it is helpful to strengthen the existing cervical cancer prevention and control programs.

## Methods and materials

The study was ethical approved by Adigrat university institutional review board with a registration number AGU/CMHS/036/09. This was conducted using unmatched case-control study design at public hospitals which have cervical cancer screening service in Tigray region. Women who are sexually active, who are with in age of 21–49 years and who live in Tigray region for the last six month were included in the study. Women who are ever screened for cervical cancer previously and in labor pain were excluded from the study. A case was women who meet the inclusion criteria and have newly screened for cervical cancer. A control was women who meet the inclusion criteria and attend gynecologic and obstetric outpatient department for any illness other than cervical cancer screening. Sample size was calculated using epi info version 7 based on the following assumptions; 95% level of confidence, 80% power, taking one to one ratio of controls to case (1:1). 11.7% proportion of 30–39 years of age among women population in Ethiopia [14]. Taking the odds ratio of being in age group of 30–39 years are 1.8 times more likely to uptake cervical cancer screening service [15]. The final sample size with 10% non-response rate was 312 cases and 312 controls.

### Sampling procedure

The hospitals were selected purposively where routine cervical cancer screening service is given and greater than one year of establishment for the screening center in Tigray region. The hospitals are; Ayder referral hospital, Mekelle general hospital, Axum St. Mary hospital and Maychew lemlem karl hospital. The total sample size was allocated using probability proportionate to size according to the proportion of average monthly client flow reviewed from registration book. Cases were selected all women screened for cervical cancer during data collection period until the required sample size was attained. After every case one control was selected from women who visit the gynecologic and obstetric outpatient department using consecutive sampling.

### Data collection procedure

Data was collected by eight trained data collectors from the client after getting informed consent; using interviewer administered structured questionnaire which includes socio demographic and economic, lifestyle and individual factors. The questionnaire was developed by reviewing similar studies [13, 15, 16], translated to Tigrigna language. Wording and consistency of the questionnaire were corrected after the pretest.

### Data analysis

The data was entered to Epi data version 3.1 then transported to SPSS version 20. Descriptive and summary statistics was used. Median was used to classify the scores of knowledge and Attitude. The median knowledge for cervical cancer and screening were 8 and 3 respectively. The median attitude was 36. Those who score of greater than the median knowledge and attitude of cervical cancer and screening assessing questions were considered knowledgeable and positive attitude respectively. Odds ratio with their 95% confidence interval and two-tailed P value was calculated and variables with P value ≤ 0.05 in the bivariate analysis was included into a multivariate logistic regression model. Statistical significance was declared if P value < 0.05.

### Ethical consideration

Ethical clearance and approval were obtained from the research and community service director of Adigrat University. Official letters was written to each health institutions from Tigray Region Health Bureau for cooperation. The respondents were informed about the objective and purpose of the study. Eventually, verbal consent of the respondents were considered regardless of their educational status. Confidentiality of the study participants was kept.

## Results

### Socio demographic characteristics of the study population

A total of 312 cases and 312 controls were participated in the study. The age distribution of the respondents shows that 121(38.8%) of the cases and 165(52.9%) of the controls were between 30–39 years of age. The mean and standard deviation of the age of cases and controls were 36.18 ± 6.86, and 32.90 ± 7.88 years, respectively. Eighty nine (28.5%) of the cases and 64(20.5%) of the controls attended college and above. At the time of this study, 232(74.4%) of the cases and 225(72.1%) of the controls were married, and 7(2.2%) of the cases and 32(10.3%) of the controls were single. More than one third 131(42.0%) of the cases and 104(33.3%) of the controls were house wife followed by governmental employee 106(34.0%) and 70(22.4%) of the cases and controls respectively. The median income per month was 2200 birr which ranges from 500 to 16700 birr and 16(5.1%) of case and 19(6.1%) of controls were have unknown income. Two hundred eighty one (90.1%) of the cases and 283 (90.7%) of the controls were identified as Orthodox Christian religion follower. In comparing of socio-demographic factors between cases of women screened for cervical cancer and controls; there was difference between cases and controls on age group, educational status, marital status and occupation (Table 1).

**Table 1:** Socio-demographic characteristics of women screened and not screened for cervical cancer in Tigray region, Ethiopia, 2018.

Variables	Cases n (%)	Controls n (%)	COR(95% CI)	P value
Age group (years)				.00
21–29	52(16.7)	121(38.8)	0.00	
30–39	165(52.9)	121(38.8)	3.21(2.22–4.63)	<0.001
40–49	95(30.4)	70(22.4)	2.24(1.43–3.50)	<0.001
Educational status				0.02
No formal education	58(18.6)	60(19.2)	0.00	
Primary education	68(21.8)	71(22.8)	0.99(0.61–1.62)	0.97
Secondary/preparatory	90(28.8)	117(37.5)	0.80(0.51–1.25)	0.32
College or above	96(30.8)	64(20.5)	1.55(0.96–2.51)	0.07
Marital status				<0.001
Single	7(2.2)	32(10.3)	0.00	
Married	232(74.4)	225(72.1)	4.71(2.04–10.90)	<0.001
Widowed	22(7.1)	23(7.4)	4.37(1.60–11.95)	<0.001
Divorced	51(16.3)	32(10.3)	7.29(2.88–18.46)	<0.001
Occupation				<0.001
House wife	68(21.8)	152(48.7)	0.00	
Private employee	109(34.9)	89(28.5)	2.74(1.84–4.08)	<0.001
Governmental/NGO employee	135(43.3)	71(22.8)	4.25(2.83–6.38)	<0.001
Income				0.99
≤ 1000	16(5.4)	16(5.5)	0.00	
1001–2000	125(42.2)	125(42.7)	1.00(0.48–2.09)	1.00
≥2000	155(52.4)	152(51.9)	1.02(0.49–2.11)	0.96
Religion				0.95
Orthodox	281(90.1)	283(90.7)	0.99(0.39–2.54)	0.99
Muslim	22(7.1)	20(6.4)	1.10(0.36–3.32)	0.87
Protestant or catholic	9(2.9)	9(2.9)	0.00	

### Reproductive and individual health related characteristic

Among the study subjects majority of them 223(71.5%) of the cases and 264(84.6%) of the controls their age at first sex were greater than 17 years old, 286(91.7%) of the cases and 248(79.5%) of the controls had ever give birth. 229(73.4%) of the cases and 215(68.9%) of the controls had ever use contraceptive, 4(1.3%) cases 2(0.6%) controls ever smoke, 295(94.6%) of the cases and 296(94.9%) of the controls had ever tested for HIV. Among the respondents 69(22.1%) of the cases and 26(8.3%) of the controls know someone with cervical cancer, and 18(5.8%) of the cases and 6(1.9%) of the controls had family history of cervical cancer. 107(34.3%) of the cases and 40(12.8%) of the controls had greater than or equals two sexual partner, 139(44.6%) of the cases and 58(18.6%) of the controls had their husband have other partner. All of the cases 312(100%) and 196(62.8%) of the controls ever heard about cervical cancer. Among the participants 121(38.8%) of the cases and 139(70.9%) of the controls had poor knowledge on cervical cancer, 119(38.1%) of the cases and 151(77.0%) of the controls had poor knowledge on cervical cancer screening. 83(26.6%) of the cases and 169(88.5%) of the controls had positive attitude on cervical cancer screening (Table 2).

**Table 2:** Reproductive and individual health related characteristics of respondent women in Tigray region, Ethiopia, 2018.

Variables	Cases n (%)	Controls n (%)	COR(95% CI)	P value
Age at first sex				<0.001
< = 16	89(28.5)	48(15.4)	0.00	
>17	223(71.5)	264(84.6)	0.46(0.31–0.68)	
Ever give birth				<0.001
Yes	286(91.7)	248(79.5)	2.84(1.74–4.62)	
No	26(8.3)	64(20.5)	0.00	
Number of birth				0.16
1–4	206(72.8)	193(78.1)	0.75(0.50–1.12)	
>4	77(27.2)	54(21.9)	0.00	
History of contraceptive use				0.22
Yes	229(73.4)	215(68.9)	0.00	
No	83(26.6)	97(31.1)	1.24 (0.88–1.76)	
STI history				<0.001
Yes	60(19.2)	32(10.3)	2.08(1.31–3.30)	
No	252(80.8)	280(89.7)	0.00	
HIV test				0.86
Yes	295(94.6)	296(94.9)	0.94(0.46–1.89)	
No	17(5.4)	16(5.1)	0.00	
HIV result				<0.001
Positive	45(15.3)	10(3.4)	5.15(2.54–10.43)	
Negative	250(84.7)	286(96.6)	0.00	
Know someone with cervical cancer				<0.001
Yes	69(22.1)	26(8.3)	3.12(1.93–5.06)	
No	243(77.9)	286(91.7)	0.00	
Family history of cervical cancer				0.02
Yes	18(5.8)	6(1.9)	3.12(1.22–7.98)	
No	294(94.2)	306(98.1)	0.00	
Number of sex partner				<0.001
> = 2	107(34.3)	40(12.8)	3.55(2.36–5.33)	
1	205(65.7)	272(87.2)	1.00	
Does your husband have other partner				<0.001
Yes	139(44.6)	58(18.6)	3.52(2.45–5.06)	
No	173(55.4)	254(81.4)	1.00	
Knowledge about cervical cancer				<0.001
Good knowledge	191(61.2)	57(24.2)	4.96(3.41–7.21)	
Poor knowledge	121(38.8)	179(75.8)	0.00	
Knowledge on cervical cancer screening				<0.001
Good knowledge	210(67.3)	59(25.0)	6.18(4.23–9.01)	
Poor knowledge	102(32.7)	177(75.0)	0.00	
Attitude on cervical cancer screening				<0.001
Positive attitude	229(73.4)	22(11.5)	21.19(12.72–35.30)	
Negative attitude	83(26.6)	169(88.5)	0.00	

### Reason for not screen before to cervical cancer

Among the respondents that knew about cervical cancer screening 69(22.1%) of cases and 56(29.3%) of controls were not screened before due to ignorance, 65(20.8%) of cases and 44(23.0%) of controls were not screened before due to low risk perception, 56(17.9%) of cases and 40(20.9%) of controls were not screened before due to perceived non-necessity (S1 Fig).

Among the respondents that knew about cervical cancer screening 210(67.3%) of cases and 121(63.0%) of controls are in nearby accessible to the screening center. Seventy three (23.4%) of case and 50(26.0%) of controls are their means of transport to cervical cancer screening center was by walk (Table 3).

**Table 3:** Accessibility of the cervical cancer screening service from the home of respondents in Tigray, Ethiopia, 2018.

Variables	Cases n (%)	Controls n (%)	COR(95% CI)	P value
Accessibility of screening center				0.61
Near	210(67.3)	121(63.0)	1.00	
Average	66(21.2)	45(23.4)	0.84(0.54–1.31)	0.45
Far	36(11.5)	26(13.5)	0.80(0.46–1.38)	0.42
Means of transport				0.50
Walk	73(23.4)	50(26.0)	1.00	
Public transport	239(76.6)	142(74.0)	1.15(0.76–1.75)	
How do you consider the cost of transport				0.07
Normal	223(93.3)	127(89.4)	1.00	
Expensive	16(6.7)	15(10.6)	0.49(0.22–1.05)	

### Factors associated with utilization of cervical cancer screening

This study tries to assess factors affecting on utilization of cervical cancer screening. Comparison of variables those tested in the bivariate logistic regression analysis were entered into multi-variable logistic regression analysis, those with p-value ≤ 0.05 and adjusted in Table 4. Controlling for the effect of other confounding factors age group of 30–39 and 40–49 years, current occupation with governmental and private employee, ever give birth, multiple sexual partner, good knowledge about cervical cancer, good knowledge and positive attitude on cervical cancer screening were found to be significantly associated with utilization of cervical cancer screening.

**Table 4:** Multi-variable analysis of selected variables with utilization of cervical cancer screening among study participants of Tigray region, Ethiopia, 2018.

Variables	Case n (%)	Control n (%)	COR (CI 95%)	AOR (CI 95%)
Age (years)				
21–29	52(16.7)	121(38.8)	0.00	0.00
30–39	165(52.9)	121(38.8)	3.21(2.22–4.63)	2.15(1.11–4.17)**
40–49	95(30.4)	70(22.4)	2.24(1.43–3.50)	3.86(1.48–10.06)**
Educational status				
No formal education	58(18.6)	60(19.2)	0.00	0.00
Primary education	68(21.8)	71(22.8)	0.99(0.61–1.62)	0.84(0.34–2.12)
Secondary/preparatory	90(28.8)	117(37.5)	0.80(0.51–1.25)	0.51(0.20–1.30)
College or above	96(30.8)	64(20.5)	1.55(0.96–2.51)	0.36(0.11–1.15)
Marital status				
Single	7(2.2)	32(10.3)	0.00	0.00
Married	232(74.4)	225(72.1)	4.71(2.04–10.90)	3.14(0.72–13.75)
Widowed	22(7.1)	23(7.4)	4.37(1.60–11.95)	0.83(0.14–5.10)
Divorced	51(16.3)	32(10.3)	7.29(2.88–18.46)	2.60(0.52–13.12)
Occupation				
House wife	68(21.8)	152(48.7)	0.00	0.00
Private employee	109(34.9)	89(28.5)	2.74(1.84–4.08)	3.85(1.87–7.92)**
Governmental/NGO employee	135(43.3)	71(22.8)	4.25(2.83–6.38)	3..17(1.31–7.66)**
Age at first sex				
< = 16	89(28.5)	48(15.4)	0.00	0.00
>17	223(71.5)	264(84.6)	0.46(0.31–0.68)	0.55(0.25–1.19)
Ever give birth				
Yes	286(91.7)	248(79.5)	2.84(1.74–4.62)	2.57(1.02–6.50)**
No	26(8.3)	64(20.5)	0.00	0.00
STI history				
Yes	60(19.2)	32(10.3)	2.08(1.31–3.30)	0.68(0.30–1.58)
No	252(80.8)	280(89.7)	0.00	0.00
HIV result				
Positive	45(15.3)	10(3.4)	5.15(2.54–10.43)	1.90(0.57–6.30)
Negative	250(84.7)	286(96.6)	1.00	1.00
Know someone with cervical cancer				
Yes	69(22.1)	26(8.3)	3.12(1.93–5.06)	0.95(0.39–2.29)
No	243(77.9)	286(91.7)	1.00	1.00
Family history of cervical cancer				
Yes	18(5.8)	6(1.9)	3.12(1.22–7.98)	0.90(0.15–5.23)
No	294(94.2)	306(98.1)	1.00	1.00
Number of sex partner				
> = 2	107(34.3)	40(12.8)	3.55(2.36–5.33)	2.65(1.10–6.39)**
1	205(65.7)	272(87.2)	1.00	1.00
Does your husband have other partner				
Yes	139(44.6)	58(18.6)	3.52(2.45–5.06)	0.89(0.41–1.94)
No	173(55.4)	254(81.4)	1.00	1.00
Knowledge about cervical cancer				
Good knowledge	191(61.2)	57(24.2)	4.96(3.41–7.21)	2.34(1.18–4.59)**
Poor knowledge	121(38.8)	179(75.8)	1.00	1.00
Knowledge on cervical cancer screening				
Good knowledge	193(61.9)	45(23.0)	6.18(4.23–9.01)	2.42(1.22–4.77)**
Poor knowledge	119(38.1)	191(77.0)	1.00	1.00
Attitude on cervical cancer screening				
Positive attitude	229(73.4)	22(11.5)	21.19(12.72–35.30)	15.10(8.01–28.44)**
Negative attitude	83(26.6)	169(88.5)	1.00	1.00

** Significantly associated with cervical cancer screening utilization

Being in the age group of 30–39 and 40–49 years were two and four times more likely to utilize of cervical cancer screening than those who were 21–29 years (adjusted odds ratio[AOR] = 2.15 95%CI:1.11, 4.17 and AOR = 3.86 95%CI:1.48, 10.06). Current occupation private and governmental employee were four and three more likely to utilize the cervical cancer screening than those house wife respectively (AOR = 3.85 95%CI: 1.87, 7.92 and AOR = 3.17 95%CI: 1.31, 7.66).

Having ever give birth was three times more likely to utilize of cervical cancer screening (AOR = 2.57 95%CI: 1.02, 6.50). Having multiple sexual partners was three times more likely to utilize of cervical cancer screening (AOR = 2.65 95%CI: 1.10, 6.40).

Good knowledge about cervical cancer and screening and positive attitude were significantly associated with utilization of cervical cancer screening (AOR = 2.34 95%CI: 1.18, 4.59), (AOR = 2.42 95%CI: 1.22, 4.77) and (AOR = 15.10 95%CI: 8.01–28.44) respectively (Table 4).

## Discussion

This study reveals that age group of 30–39 and 40–49 years, current occupation with private and governmental/ NGO employee, having other sex partner and ever give birth, good knowledge about cervical cancer, good knowledge and positive attitude on cervical cancer screening were predictors of utilization of cervical cancer screening.

The study subjects have difference in age group of 30–39 and 40–49 years between cases and controls as compared to the age group of 21–29 years. Being in the age group of 30–39 and 40–49 years was significantly associated with utilization of cervical cancer screening service. Though most of the cases are in the age group of 30–39 and 40–49 years there are still cases in the age group of 21–29 years. This is consistent with study in Mekelle, Ethiopia, which shows that women in the age range of 30–39 years were about 1.80 times more likely to be screened for cervical cancer compared with those 21–29 years old [15]. It was also similar to study finding in Tanzania, age groups of 40–49 years had significantly higher odds of being screened [17]. This is similar with the study conducted in Addis Ababa, Ethiopia, which reported that willingness and acceptance of screening were being in age group of 40–49, 50–59 and >60 years as compared below 29 years [18]. The odds of ever screening was eight times higher for those whose age is ≥30 years than those whose age is <30 years [16]. This study is consistent with other studies that show older age of participants was also associated with higher risk of cervical cancer compare to below the age of 30 [19].

Current occupation with private and governmental or nongovernmental employee women were more likely to be screened as compared with housewives. This is consistent with study conducted in Ghana, shows there is statistically significant relationship between employed participants and cervical cancer screening utilization [20]. Study in Nigeria shows occupation were significantly associated with awareness of cervical cancer and screening [21]. Among HIV positive women in Ghana shows employment status more likely to have intention to screen [22]. Study conducted in Addis Ababa shows good knowledge about cervical cancer among governmental and nongovernmental organization employees were two times higher than among unemployed participants [23]. This may be due to having exposure to health information among those employees.

In this study women who ever give birth were utilized the cervical cancer screening services. This is similar with study conducted among teachers in Tanzania which shows multiparous were 3 times more likely to utilize the service compared to those with zero parity [24]. A study from Ghana there is significant relationship between number of children and cervical cancer screening utilization of the participants [20]. Those women who have <5 children were 79% less likely to be screened than those women who had ≥ 5 children [16]. This is assumed that previous pregnancies of a woman may expose her to receive health education and particularly on sexual and reproductive health many times compared to those with no previous pregnancies.

It is found that women with history of multiple sexual partners were more likely to utilize cervical cancer screening service. This is consistent with study conducted in Mekelle, women who have history of multiple sexual partners were 1.64 times more likely to undergo screening when compared to those who have no history of multiple sexual partner [15]. Participants in Tanzania shows with more than one lifetime sexual partners were found to be two times more likely to utilize cervical cancer screening services compared to those with one lifetime sexual partner [24]. Study conducted in Yirgalem General Hospital, Ethiopia shows multiple sexual partners are significantly associated with knowledge of cervical cancer as compared to those women who have no exposure of multiple sexual partners [25]. That can be explained by the fact that women with many lifetime partners may perceive themselves to be more at risk than the others.

Study conducted in Arba Minch, Ethiopia shows knowledge of cervical cancer was found to be significantly associated factor for cervical cancer screening [26]. In Tanzania knowledge of cervical cancer were strongly associated with being screened [17]. In Mekelle, Ethiopia among age Eligible women, knowledge on cervical cancer screening was 2.36 times more likely to undergo screening when compared to those who were not knowledgeable [15]. Study conducted among nurses in Mekelle, Ethiopia shows the odds of getting screened for cervical cancer have a positive attitude 3.4 times as compared to nurses who have negative attitudes [13]. Respondents who had negative attitude had 63% lesser odds of being screened compared to those who had positive attitudes towards screening in Nigeria [27]. Those studies were consistent with this study finding.

## Conclusions

In this study, women with the age group of 30–39 and 40–49 years, current occupation with governmental and private employee, having lifetime multiple sexual partners and ever give birth were more likely to utilized cervical cancer screening. Good knowledge about cervical cancer, good knowledge and positive attitude about cervical cancer screening were significantly associated with utilization of cervical cancer screening.

## Recommendations

There is a need to strength policy and guidelines on cervical cancer screening among women particularly with regarding occupation, number of sexual partners and parity. Health professional should give advice to women with in age group of 21–29 years, current occupation as housewife and women with single sexual partner to be screened for cervical cancer. All stakeholders, particularly the health sector, should give priority on the promotion and initiation of women to acquired good knowledge and positive attitude on cervical cancer screening.

## Limitations

Studies should be done regarding readiness of cervical cancer screening service, quality of service, provider knowledge and other related factors.

## Supporting information

S1 Fig Reason for not screened before in study participants in Tigray region, Ethiopia, 2018, (A) Case and (B) Control. (TIF)

S1 File Questionnaire English version. (PDF)

S2 File Questionnaire in local language. (PDF)

S3 File Data set. (SAV)
