# Supplementary figures and images for: Factors affecting utilization of cervical cancer screening services among women attending public hospitals in Tigray region, Ethiopia, 2018; Case control study
Source: PLoS One. 2019 Mar 14;14(3):e0213546. doi: 10.1371/journal.pone.0213546 (PMC6417770; doi:10.1371/journal.pone.0213546)

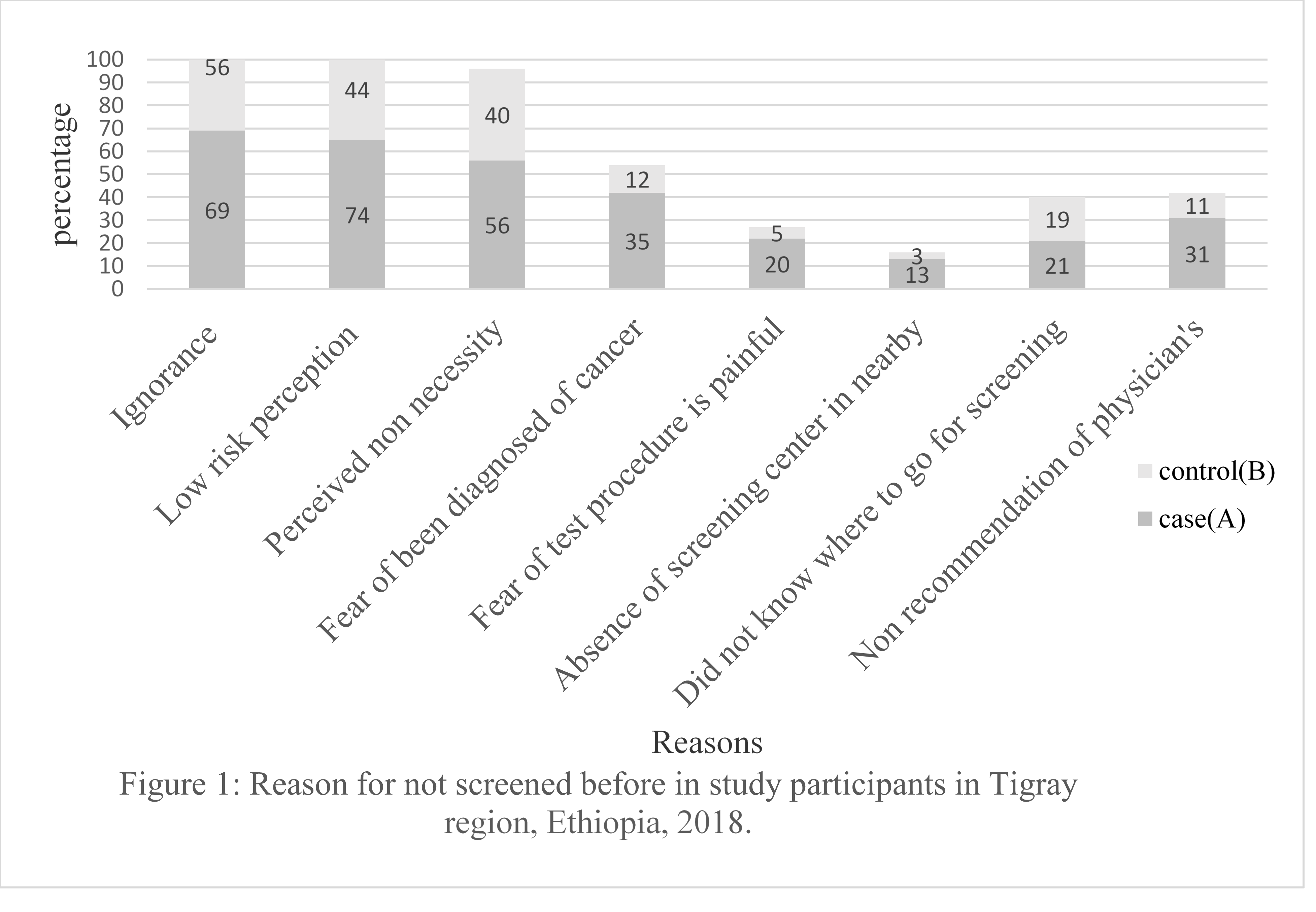

Supplement: S1 Fig — (TIF) [file pone.0213546.s001.tif]
