# Supplementary material for: Factors affecting utilization of cervical cancer screening services among women attending public hospitals in Tigray region, Ethiopia, 2018; Case control study
Source: PLoS One. 2019 Mar 14;14(3):e0213546. doi: 10.1371/journal.pone.0213546 (PMC6417770; doi:10.1371/journal.pone.0213546)
Supplement: S1 File — (PDF) [file pone.0213546.s002.pdf]

Questionnaire (English Version)

Adigrat University College of Medicine and Health Sciences Department of Public Health information sheet on the factors affecting utilization of cervical cancer screening among women attending in public hospitals in Tigray region.

Hello! How are you? My name is __________. Now I am a research team member to be conducted here by Adigrat university, supervised by department of Public Health instructors. The purpose of the study is assessing the factors affecting utilization of cervical cancer screening services among women attending in public hospitals in Tigray region. If you agree to participate in the study as respondent, you will not have any risk in participating in the study except the time you spent during the interview. The study may be advantageous in identifying factors affecting utilization of cervical cancer screening services, so it is important to develop strategies that help to improve the prevention and control methods of cervical cancer. All the genuine information obtained from you will be strictly kept confidential, your participation is surely voluntary, and no monetary incentives will be given for your participation in the study. You can withdraw any time during conducting the study, also your participation, non-participation, or refusal to answer questions will not have any effect on your life, and your name will not be recorded on this form. If you have any question Mrs Hirut Teame is the contact person. Hirut can be reached through a call at 0946415072.

Are you willing to participate in the interview and stay with us for few minutes (15-20) now?

[ ] Yes, Go to next page [ ] No, Thanks! Proceed to next eligible participant

Note: Women who are sexually active, who are above the age of 30 years and who live in Tigray region for the last six month.

Consent form

I am informed that my identity and the information I give will be treated confidentially. I have also been informed that I can refuse to participate in the study or not to respond to questions if I am not interested. Furthermore, I have been informed that I can stop responding to the questions at any time in the process. I am informed that my participation, non-participation, or refusal to answer questions will not have any effect on my life. I am informed that no monetary incentives will be given for my participation in the study. I am also informed that my response will be used to develop strategies that help to improve the prevention and control methods of cervical cancer.

If the study subject agrees to participate in the study, thank her and start the interview.

Interviewer signature certifying that informed consent has been given verbally by the respondent.
Interviewer‘s
Name_____________________Signature_________________Date_______________

Note: No need of enforcing the clients to be included in the study.

1.1. Date of interview……………………….

1.2. Study site……………………..

1.3. Code of the interview …………………………

Thank you!

General information

For each question, make a circle around the number that corresponds to the answer; fill the blanks with the answer or mark “x”.

1. Participant’s code number: ____________

Women screened for cervical cancer ☐ **women not screened for cervical cancer** ☐

Part 1: Socio-demographic characteristics

Ser. No	Question	Response	Skip
1.1	How old are you? (completed years)	___________	
1.2	What is your level of education?	Don’t write and read…………………..1<br>Only read and write……………………2<br>Primary(1-4)……………………………3<br>Primary (5-8)…………………………..4<br>Secondary(9-10)………………………..5<br>Preparatory or technical or vocational…6<br>Diploma…………………………………7<br>Higher(bachelor degree and above)…….8	
1.3	What is your marital status?	Single………………1<br>Married……………..2<br>Widowed…………….3<br>Divorced………………4	
1.4	How old were you when you first marriage? (If she is already married once)	______________	
1.5	What is your current occupation status?	House wife………………1<br>Farmer……………………2<br>Merchant………………….3	

		Daily laborer………………4<br>Governmental employee……5<br>Private/NGO employee…….6<br>A. Others (specify__________)…..7	
1.6	How much is your family average monthly income(ETB)	Total _______________<br>Unknown……………….1	
1.7	What is your religion?	Orthodox Christian………………..1<br>Muslim……………………………..2<br>Protestant…………………………....3<br>Catholic………………………………4<br>Others(specify__________)…………..5	
1.8	Ethnicity	Tigray…………………1<br>Amhara……………….2<br>Others…………………3	

Part II: Individual related factors

2.1.	Age at first sexual intercourse in years	_________In years	
2.2.	Have you ever been give birth?	Yes…………………1<br>No…………………..2	
2.3.	If answer for Q 2.2 yes, How many times?	___________	
2.4.	Did you ever use any contraceptive methods	yes............1<br>No.............2	If no skip to 2.8
2.5.	If yes what type?	Oral contraceptive pills..........1<br>Injectable...............................2<br>Norplant ....3<br>IUCD....................................4<br>Other specify 5	
2.6.	If the response to the above question is 1, for how long did you use oral contraception?	_________Years	
2.7.	Are you currently using oral contraception?	Yes..................1<br>No...................2	
2.8.	Do you smoke?	Yes..................1<br>No...................2	
2.9.	Have you had a sexually transmitted infection in your lifetime?	Yes..................1<br>No...................2	
2.10.	Have you been tested for HIV before?	A. Yes B. No	
2.11.	If answer for Q 2.10 yes, what was the result?	A. Positive B. Negative<br>C. Unknown	
2.12.	If answer for Q 2.11 positive, did you start antiretroviral therapy?	A. Yes B. No	
2.13.	Do you know someone with cervical cancer	Yes …………..1<br>No……………2	
2.14.	Do you have family (mother or sister) history of cervical cancer?	Yes……………1<br>No…………….2	
2.15.	How many sexual partners have you had in your lifetime?	___________	

2.16.	Does your partner have other partners?	Yes…………1<br>No……………..2	

Part III: Questions on knowledge about cervical cancer and screening

3.1.	Have you ever heard about cervical cancer?	Yes............1<br>No..............2	If no, end the here
3.2.	Where did you first learn about cervical cancer<br>Multiple answer possible	News Media.................................1<br>Brochures, posters and other printed materials...........2<br>Health workers.................................3<br>Family, friends, neighbors and colleagues..................4<br>Religious leaders................................5<br>Teachers...6<br>Other (please explain)..........................7	
3.3.	What are the symptoms of cervical cancer?<br>Multiple answer possible	Vaginal bleeding.................................1<br>Foul smelling of vaginal discharges......2<br>Contact bleeding……………………….3<br>Do not know..4<br>Other..5	
3.4.	What are the risk factors for cervical cancer?<br>Multiple answer possible	Acquiring HPV virus............................1<br>Having multiple sexual partners...........2<br>Early sexual intercourse.......................3<br>Give birth to many children.....................4<br>Using oral contraceptive for long time..........5<br>Cigarette smoking...............................6<br>HIV/AIDS……………………………7<br>Do not know…………………………8<br>Other specify 9	
3.5.	Who vulnerable to cervical cancer	Women>50 years of age……………...1<br>Reproductive age ………….............2<br>Both …………………………………3<br>Do not know………………………4	

3.6.	How can a person prevent getting cancer of the cervix?<br>Multiple answer possible	through vaccination of HPV vaccine..........1<br>Avoid multiple sexual partners...................2<br>Avoid early sexual intercourse....................3<br>Avoid give birth to many children...................4<br>Avoid using oral contraceptives<br>for long time...5<br>Quit smoking...6<br>Screening for cervical cancer.....................7<br>Do not know..8<br>Other (please explain).................................9	
3.7.	Can cancer of the cervix be cured in its earliest stages?	Yes..........................1<br>No ....2<br>Don’t know 3	
3.8.	How can someone with cancer of the cervix be treated? Multiple answer possible	Herbal remedies...................................1<br>Surgery...2<br>Specific drugs given by hospital...........3<br>radiotherapy..4<br>Do not know..5<br>Other(please explain)…………………6	
3.9.	How expensive do you think cancer of the cervix treatment is in this Country	It is free of charge...................1<br>It is reasonably priced.............2<br>It is somewhat/moderately<br>expensive................................3<br>It is very expensive.................4<br>Don't know...............................5<br>other(specify) 6	
3.10.	Are there screening procedures to detect premalignant cervical lesion?	Yes.............1<br>No...............2	If no end here
3.11.	Do you heard of benefits of screening	Yes……………1<br>No…………….2	

3.12.	How frequent is screening for premalignant cervical lesion done?	Once every year...................1<br>Once every three years.........2<br>Once every 5 years...............3<br>Don't know.........................4<br>Any other(mention).............5	
3.13.	Who should be screened?	Women of 25 years and above....................................1<br>Prostitutes..2<br>Elderly women...3<br>Don't know...4<br>Other..5	
3.14.	Do you know procedures used in screening for premalignant cervical lesions?	Yes..................1<br>No...................2	
3.15.	Can you mention any of the procedures used in screening for premalignant cervical lesions?<br>Multiple answer possible	VIA..................1<br>VILI.................2<br>Pap Smear........3<br>don't know........4<br>other…………5	

Part IV: Attitude Questions

4.1.	Cervical cancer is highly prevalent in our country and is a leading cause of deaths amongst all malignancy in Ethiopia.	Strongly disagree......................................1<br>Disagree..2<br>Neither agree nor disagree.........................3<br>Agree……..4<br>Strongly agree……...................................5	
4.2.	Any adult woman including you can acquire cervical cancer	Strongly disagree......................................1<br>Disagree..2<br>Neither agree nor disagree.........................3<br>Agree……..4<br>Strongly agree……...................................5	
4.3.	Cervical cancer is a killer if not detected early	Strongly disagree......................................1<br>Disagree..2	

		Neither agree nor disagree.........................3<br>Agree……...4<br>Strongly agree……..................................5	
4.4.	Cervical cancer cannot be transmitted from one person to another	Strongly disagree......................................1<br>Disagree..2<br>Neither agree nor disagree.........................3<br>Agree……...4<br>Strongly agree……..................................5	
4.5.	Screening for premalignant cervical lesions helps in prevention of cervical cancer	Strongly disagree......................................1<br>Disagree..2<br>Neither agree nor disagree.........................3<br>Agree……...4<br>Strongly agree……..................................5	
4.6.	Screening causes no harm to the client	Strongly disagree......................................1<br>Disagree..2<br>Neither agree nor disagree.........................3<br>Agree……...4<br>Strongly agree……..................................5	
4.7.	Screening for premalignant cervical lesions is not expensive	Strongly disagree......................................1<br>Disagree..2<br>Neither agree nor disagree.........................3<br>Agree……...4<br>Strongly agree……..................................5	
4.8.	If screening is free and causes no harm, will you screen	Strongly disagree......................................1<br>Disagree..2<br>Neither agree nor disagree.........................3<br>Agree……...4<br>Strongly agree……..................................5	
4.9.	It is not embarrassing to go through screening procedure	Strongly disagree......................................1<br>Disagree..2	

		Neither agree nor disagree........................3<br>Agree……...4<br>Strongly agree……..................................5	
4.10.	If you want to get a screening, will you allow male doctors to examine your cervix	Strongly disagree......................................1<br>Disagree..2<br>Neither agree nor disagree........................3<br>Agree……...4<br>Strongly agree……..................................5	
4.11.	If you develop cervical cancer, you will consult a doctor without being scared	Strongly disagree......................................1<br>Disagree..2<br>Neither agree nor disagree........................3<br>Agree……...4<br>Strongly agree……..................................5	

Part V: Questions related to not screened for cervical cancer before

5.1.	What is the reason not screened before?	Ignorance………………………1<br>Low risk perception…………….2<br>Perceived non necessity ………..3<br>Husband did not agree…………..4<br>High cost of screening…………..5<br>Fear of been diagnosed of cancer ……..6<br>Fear of test procedure is painful………..7<br>Did not know where to go for screening…..8<br>Absence of screening centers in nearby…..9<br>Faith in God………………………………10<br>Physician's non recommendation…………11<br>Others(specify) ______________________	
5.2.	did you involve spouse in making decision for screening	Yes………………..1<br>No…………………2	

Part VI: ACCESSIBILITY OF THE SCREENING SERVICE

6.1.	Have you heard about where the screening center is?	Yes …………1<br>No…………..2	
6.2.	How far is the screening center from where you are living?	_________________KM or<br>Very near…………..1<br>Near………………..2<br>Normal…………….3<br>Far…………………4<br>Very far …………...5	
6.3.	What is the means of transport from your home to the screening center?	Walk…………………1<br>Public transport……….2<br>Others………………3	
6.4.	If by public transport, how much do you spend for transport from your home to the Facility?	_________________birr	
6.5.	How do you consider the cost of transport to the facility?	Normal…………1<br>Expensive………2	
