# Supplementary material for: Factors affecting utilization of cervical cancer screening services among women attending public hospitals in Tigray region, Ethiopia, 2018; Case control study
Source: PLoS One. 2019 Mar 14;14(3):e0213546. doi: 10.1371/journal.pone.0213546 (PMC6417770; doi:10.1371/journal.pone.0213546)
Supplement: S2 File — (PDF) [file pone.0213546.s003.pdf]

ዓዲግራት ዩኒቨርሲቲ

ናይ ሕክምናን ጥዕና ሳይንስ ኮሌጅ

ናይ ሕብረተሰብ ጥዕና ኣተሓሳልዋ ትምህርቲ ክፍሊ

ንደቂ ኣንስቲዮ ነባር ክልል ትግራይ ብዛዕባ ኣብ ፀገም ኣጠቃቅማ ናይ ማህፀን ኣፍ ካንሰር ምርመራ መበገሲታት ዝምልከት ዝተዳለወ መሕትት ፡፡

ጥዕና ይሃብለይ፤

ኣነ.......................................ኣብ ዓዲግራት ዩኒቨርሲቲ ናይ ሕብረተሰብ ጥዕና ኣተሓሳልዋ ትምህርቲ ክፍሊ ኣባል ኮይነ ሕዚ ብዛዕባ ናይ ደቂ ኣንስቲዮ ፍልጠት ፣ጵልየትን ፣ተግባር(ኣጠቃቅማ) ናይ ማህፀን ኣፍ ካንሰር ኣብ ለ ትግራይ ሰሜናዊ ኢትዮጵያ ንመማልኣ ናይ መጀመሪያ ዲግሪ ናይ ሕብረተሰብ ጥዕና ኣተሓሳልዋ መፅናዕታዊ ፅሑፍ ያካየድ ኣለኹ፡፡ ጥቅሚ ናይዘ መፅናዕታዊ ፅሑፍ በዛዕባ ናይ ማህፀን ኣፍ ካንሰር ፍልጠት ፣ጵልየትን ኩነታት ኣጠቃቅማ ንምልካዕ ኮይኑከይዲ ናይ እዚ መፅናዕታዊ ጽሑፍ ኣብ ናይ ክትትልን ቁፅፅርን ገምጋሚ እዩ ዝካየድ እዚ መሕተት ዕዚ ብቃል ኮይኑ እዚ መሕተት ብእንግሊዝኛን ብትግርኛን እዩ ዝተዳለወ፡፡

እዚ ብፈቃደኝነት ዝተመሰረተ መሕተት ካብ 30-40 ደቂቃ ዝወስድ ኮይኑ ኣብዚ መጠይቅ ናይ ዘይምስታፍ መሰሎም ዝተሓለወ እዩ ብተወሳኺ ምስታፍ ጀሚሮም ናይ ምቁራፅ መሰል ኣለኹም፡፡

ነዚ መሕተት ንዝህብዎ ምላሽ ብሙሉእ ምስጢራዊነቱ ዝተሓለወ ስለዝኮነ ምንም ዓይነት ስግኣት ክኣትዎም የብሉን ስለዝኮነ ኣብዚ መሕተት ክሳተፉ ብኽብሪ ንሓትት፡፡

ኣብዚ መፅናእታዊ ጽሑፍ ንምስታፍ ፍቃደኛ ድዮም ?

እወ____________________

ኣይኮንኩን________________

ንምስታፍ ፈቃደኛ እንተኾይኖም ናብ ዝቀፅል ገፅ ስገር/ሪ

ሓበሬታ፡- ኣብዚ ፅንዓት ክትሳተፍ ዘለዋ ኣዶ

- ቅድሚ ሐዚ ናይ ማህፀን ኣፍ ካንሰር ምርመራ ተመርሚራ ዘይትፈሊጥ ክትኮን ኣለዋ፡፡
- ጥንስቲ ዘይኮነት ኣዶ 21-50 ዕድመ ዝኮነት ኣዶ

ናይ ኣፍ ማህፀን ካንሰር ምርመራ ዝገበረት ☐ ናይ ኣፍ ማህፀን ካንሰር ምርመራ ዘይገበረት ☐

1. ኣጠቃላሊ ማሕበራዊ መረዳእታ ዝተመልከተ መሕተት

ተ.ቁ	ሕቶታት	መልሲ
1.1	ዕድመ	____________________________
1.2	ደረጃ ትምህርቲ	1. ምፅሓፍ ምንባብ ዘይትክእል 2. ምፅሓፍ ምንባብ ዝክእል<br>3. ካብ 1-4 ክፍሊ 4. .ካብ 5-8 ክፍሊ 5. ካብ 9-10 ክፍሊ<br>6.ካብ 11-12 ክፍሊ 5. ዲፕሎማ 7. ቀዳማይ ዲግሪን ካብኡ ንላዕሊን
1.3.	ኩነታት ሓዳር	1. ዘይተመረዐወት 2. ዝተመረዐወት<br>3. ሰብኣያ ዝሞታ 4. ዝተፋተሓት
1.4.	ኣብ ክንደይ ዓመትኺ ተመርዒዊኺ? (እንድሕር ዝተመርዐወት ኮይና)	________
1.5	ስራሕ	1. የቤት እመቤት 2. ሓረስታይ 3. ነጋዴ<br>4. ናይ ማዓልቲ ሰራሕተኛ 5. ሰራሕተኛ መንግስቲ<br>6. ናይ ውልቓ ስራሕ (ዘይመንግስታዊ ስራሕ) 8.ካሊእ _______________
1.6	ወርሓዊ እቶት(ብብር)	1_________________ 2. ኣይፈልጦን
1.7	ሃይማኖት	1. ኦርቶዶክስ ተዋህዶ 2. ሙስሊም 3. ፕሮቴስታንት<br>4. ካቶሊክ 5.ካሊእ_________________
1.8	ብሄር	1. ትግራይ 2. ኣምሓራ 3.ካሊእ_____________

2. ተጋሊፂነት ናይ ኣፍ ማህፀን ካንሰር (ምንቀርሳ) ኣብ ሞንጎ ተሳተፍቲ እዚ መፅናእታዊ ጽሑፍ

ተ.ቁ	ሕቶታት	መልሲ
2.1.	ኣብ ክንደይ ዓመትኺ ፆታዊ ርክብ ጀሚርኺ?	_______________________
2.2.	ወሊድኺ ዶ ትፈልጢ?	1. እወ 2. ኣይፈልጥን
2.3.	መልሲ ንተ.ቁ 2.2 እወ እንተኮይኑ ክንደይ ግዜ?	_________________
2.4.	መከላከሊ ጥንሲ ትጠቂምኺ ዶ ትፈልጢ?	1. እወ 2. ኣይፈልጥን

2.5.	መልሲ ንተ.ቁ 2.4 እወ እንተኮይኑ እንታይ ዓይነት መከላከሊ ጥንሲ ትጥቀሚ? (ካብ ሓደ ንላዕሊ ትመርፂ ትኽእሊ ኢኺ)	1. ክኒና 2. ብመርፍእ ዝውሰድ 3. ኣብ ትሕቲ ቆርበት ዝቅበር 4. ኣብ ማህፀን ዝኣቱ 5. ካልእ _______
2.6.	መልሲ ንተ.ቁ 2.5 ክኒና እንተኮይኑ ንክንደይ ዓመት ክኒና ተጠቒምኺ?	_______________ ዓመት
2.7.	ኣብ ከዚ ግዜ ዝዋሓጥ ክኒን ትወስዲ ዲኺ?	1. እወ 2. ኣይወስድን
2.8.	ሽጋራ ተትክኺ ዲኺ?	1. እወ 2. ኣይትክክን
2.9.	ብፆታዊ ርክብ ዝመሓላለፍ ሕማም ሓሚምኺ ትፈልጢ ዶ?	1. እወ 2. ኣይፈልጥን
2.10.	ናይ ኤች.አይ.ቪ ምርመራ ተመርሚርኺ ዶ ትፈልጢ?	1. እወ 2. ኣይተመርመርኩን
2.11.	መልሲ ንተ.ቁ 2.10 እወ፣ እንተኮይኑ፣ ውፅኢቱ እንታይ ነይሩ?	1. ኣለኒ 2. የብለይን 3. ኣይፍለጥን
2.12.	መልሲ ንተ.ቁ 2.12 ኣለኒ እንተኮይኑ፣ ናይ ፀረ ኤች. አይ. ቪ መድሓኒት ጀሚርኺ ዶ?	1. እወ 2. ኣይጀመርኩን
2.13.	ብናይ ኣፍ ማህፀን ካንሰር ዝተትሓዘ ሰብ ትፈልጢ ዶ?	1. እወ 2. ኣይፈልጠን
2.14.	ብ ናይ ማህፀን ኣፍ ካንሰር ዝተተሓዝት ቤተሰብ (ኣዶኺ ወይም ሓፍትኺ) አለኺ ዶ?	1. እወ 2. ኣብለይን
2.15.	ኣብ ሂወትኺ ክንደይ ስብኣይ ኔሮምኺ?	________
2.16.	ስብኣይኺ ካልኦት ኣንስቲ ኣለዎኦ ዶ?	1. እወ 2. የብሉን

3. **ናይ ኣፍ ማህፀን ካንሰር ኣፋልጦ ብተመልከት**

ተ.ቁ	ሕቶታት	መልሲ
3.1.	ብዛዕባ ናይ ኣፍ ማህፀን ካንሰር ኣንቢብኪ ፣ ርኢኪ፣ ሰሚዕኪ፣ ትፈልጢ ዶ?	1. እወ 2. ኣይፈልጥን
3.2.	እቲ ሓበሬታ ናይ ኣፍ ማህፀን ካንሰር ካብ ኣይናይ ፍልፍል ረኪብኪዮ? (ካብ ሓደ ንላዕሊ መልሲ ይካኣል እዩ)	1. ቴሌቪዥንን ሬዲዮ 2. መጽሔት ፣ ጋዜጣ ፣ ፖስተር 3. በዓል ሞያ ጥዕና 4. ኣባል ቤተሰብን ጎረቤትን አርኪ 5. ናይ ሃይማኖት መራሕቲ 6. መምህር 7. ካሊእ___________________
3.3.	ናይ ኣፍ ማህፀን ካንሰር ምልክታት እንመን እዮም? (ካብ ሓደ ንላዕሊ መልሲ ይካኣል እዩ)	1. መደመይቲ ካብ ሞንጎ ወርሓዊ ጽጌያት 2. ሕማቅ ጨና ዘለዎ ናይ ማህፀን ፈሳሲ 3. መደመይቲ ድሕረ ፆታዊ ርክብ 4. ኣይፈልጥን 5.ካሊእ_____________

3.4.	ናይ ኣፍ ማህፀን ካንሰር ከቃልዑ ዝኽእሎ ነገራት?<br>(ካብ ሓደ ንላዕሊ መልሲ ይካኣል እዩ)	1. ን HPV ምግላፅ 2. ካብ ሓደ ሰብኣይ ንላዕሊ ፆታዊ ርክብ ብምግባር<br>3. ትሕቲ ዕድመ ፆታዊ ርክብ ብምግባር 4. ብዙሓት ቖልዑ ምውላድ<br>5. ናይ ጥንሲ መከላከሊ ከኒን 6. ሽጋራ ምትካክ<br>7. HIV/AIDS 8. ኣይፈልጥን 9. ካሊእ________________________
3.5.	ናይ ኣፍ ማህፀን ካንሰር ዝቃልዑ ንንመን እየን?	1. ካብ 50 ዓመት ንላዕሊ ዝኮና 2. ኣብ ክልል ዕድመ ምውላድ ዘለዋ<br>3. ኣብ ክልቲኡ ክልል ዕድመ ዘለዋ 4. ኣይፈልጦን
3.6.	ከመይ ገይረና ናይ ኣፍ ማህፀን ካንሰር ንከላከል?<br>(ካብ ሓደ ንላዕሊ መልሲ ይካኣል እዩ)	1. HPV ክትባት ምዉሳድ 2. ካብ ምስ ሓደ ሰብኣይ ንላዕሊ ፆታዊ ርክብ ዘይምግባር<br>3. ትሕቲ ዕድመ ፆታዊ ርክብ ዘይምግባር 4. ብዙሓት ቖልዑ ዘይምውላድ<br>5. ናይ ጥንሲ መከላከሊ ከኒን ዘይምውሳድ 6. ሽጋራ ዘይምትካክ<br>7. ናይ ኣፍ ማህፀን ካንሰር ምርመራ ብምግባር 8. ዘይፈልጥ<br>9. ካሊእ______________________
3.7.	ቀልጢፍኪ እንተተሓከምኪ ናይ ኣፍ ማህፀን ካንሰር ክድሕን ይክእል ዶ?	1. እወ 2. ኣይድሕንን 3. ኣይፈልጥን
3.8.	ሓንቲ ናይ ኣፍ ማህፀን ካንሰር ተጠቃዒት ብከመይ መንገዲ ትሕከም? (ካብ ሓደ ንላዕሊ መልሲ ይካኣል እዩ)	1. ናይ ባህሊ ሕክምና 2. መጥባሕቲ 3. ኣብ ሆስፒታል ዝወሃብ መድሓኒት<br>4. ናይ ጨረር ምርመራ 5. ኣይፈልጥን 6.ካሊእ___________________
3.9.	ዋጋ ናይ ማህፀን ካንሰር ሕክምና ከመይ ትሪእኦ?	1. ነፃ እዩ 2. ተመጣጣኒ ዋጋ 3. ብመጠኑ ክባር እዩ<br>4. ክባር እዩ 5. ኣይፈልጥን 6.ካሊእ_______________
3.10.	ን ኣፍ ማህፀን ካንሰር ቅድመ ካንሰር ምርመራ ኣለዎ ዶ?	1. እወ 2. ኣይፈልጥን
3.11.	ናይ ኣፍ ማህፀን ቅድመ ካንሰር ምርመራ ጥቕምታት ሰሚዕኺ ዶ ትፈልጢ?	1. እወ 2. ኣይፈልጥን
3.12.	ናይ ኣፍ ማህፀን ቅድመ ካንሰር ምርመራ በበከንደይ ጊዜ ክግበር ኣለዎ? (ካብ ሓደ ንላዕሊ መልሲ ይካኣል)	1. ሓደ ጊዜ ኣብ ዓመት 2. ሓደ ጊዜ ኣብ 3 ዓመት 3. ሓደ ጊዜ ኣብ 5 ዓመት<br>4. ኣይፈልጥን 5. ካሊእ___________________________

3.13.	ናይ ኣፍ ማህፀን ቅድመ ካንሰር ምርመራ ኣብ ኣይናይ ጉጅለ ደቂ ኣንስቲዮ ክግበር ኣለዎ? (ካብ ሓደ ንላዕሊ መልሲ ይካኣል እዩ)	1. 25 ዓመትን ካብኡ ንላዕሊን ዝኾና ደቂ ኣንስቲዮ 2. ነብሰን ሸይጠን ዝናበራ ደቂ ኣንስቲዮ 3. ዓበይቲ ደቂ ኣንስቲዮ 4. ኣይፈልጥን 5. ካልኦት ________________________
3.14.	ናይ ኣፍ ማህፀን ቅድመ ካንሰር ምርመራ ከመይ ኣይነት ምርመራ ከምዝካየድ ትፈልጢ ዶ?	1. እወ 2. ኣይፈልጥን
3.15.	ናይ ቅድመ ካንሰር ምርመራ ብከመይ መንገድ ይካየድ? (ካብ ሓደ ንላዕሊ መልሲ ይካኣል እዩ)	1. VIA 2.VIU 3. PAP Smear 4. ኣይፈልጥን 5.ካሊእ____________________

4. ኣመለካኸታ ናይ ኣፍ ማህፀን ቅድመ ካንሰር ምርመራ ዝምልከት

ተ.ቁ	ሕቶታት	መልሲ
4.1.	ናይ ኣፍ ማህፀን ካንሰር ኣብ ኢትዮጵያ እቲ ዝበዝሐ ናይ ካንሰር ሞት ፣ ዋና ናይ ሕ/ሰብ ጥዕና ፀገም ድዩ?	1. ብጣዕሚ ኣይስማዕማዕን 2. ኣይስማማዕን 3. ሓሳብ የብለይን 4. ተሰማሚዐ 5. ብጣዕሚ ይስማዕማዕ
4.2.	ዝኮነት መንእሰይ ጓል ኣንስተይቲ ንዐኪ ወሲኩን ናይ ኣፍ ማህፀን ካንሰር ክሕዘክን ይከኣል ዶ ?	1. ብጣዕሚ ኣይስማዕማዕን 2. ኣይስማማዕን 3. ሓሳብ የብለይን 4. ተሰማሚዐ 5. ብጣዕሚ ይስማዕማዕ
4.3.	ናይ ኣፍ ማህፀን ካንሰር ቀዲሙ እንተዘይተመርሚሩ ንሞት የጋልፅ ዶ?	1. ብጣዕሚ ኣይስማዕማዕን 2. ኣይስማማዕን 3. ሓሳብ የብለይን 4. ተሰማሚዐ 5. ብጣዕሚ ይስማዕማዕ
4.4.	ናይ ኣፍ ማህፀን ካንሰር ካብ ሓደ ሰብ ናብ ሓደ ሰብ ከሓልፋ ኣይክኣልን ?	1. ብጣዕሚ ኣይስማዕማዕን 2. ኣይስማማዕን 3. ሓሳብ የብለይን 4. ተሰማሚዐ 5. ብጣዕሚ ይስማዕማዕ
4.5.	ቅድሚ ምልክታት ምርኣዮም ናይ ኣፍ ማህፀን ካንሰር ምርመራ ብምግባር ናይ ኣፍ ማህፀን ካንሰር ምክልኻል ይካኣል ኢልኪ ታኣምኒ ዶ?	1. ብጣዕሚ ኣይስማዕማዕን 2. ኣይስማማዕን 3. ሓሳብ የብለይን 4. ተሰማሚዐ 5. ብጣዕሚ ይስማዕማዕ
4.6.	ምፍታሽ/ምምርማር ንተገልጋለይ /ዓሚል ጉድኣት የብሉን ?	1. ብጣዕሚ ኣይስማዕማዕን 2. ኣይስማማዕን 3. ሓሳብ የብለይን 4. ተሰማሚዐ 5. ብጣዕሚ ይስማዕማዕ
4.7.	ምፍታሽ /ምምርማር ናይ ኣፍ ማህፀን ካንሰር ክባር ኣይኮነንን ?	1. ብጣዕሚ ኣይስማዕማዕን 2. ኣይስማማዕን 3. ሓሳብ የብለይን 4. ተሰማሚዐ 5. ብጣዕሚ ይስማዕማዕ

4.8.	እንድሕር ምምርማር ነፃ እና ምክንያታዊ ጉድኣት ዘይብሉ ምርመራ ክትገብሪ ትኽዕሊ ዶ?	1. ብጣዕሚ ኣይስማዕማዕን 2. ኣይስማማዕን<br>3. ሓሳብ የብለይን 4. ተሰማሚዐ 5. ብጣዕሚ ይስማዕማዕ
4.9.	እንድሕር ናብ ኣፍ ማህፀን ቅድመ ካንሰር ምርመራ ደሊኺ ተባዓታይ ዶክተር ክምርምረኺ ትፈቕዲ ዶ?	1. ብጣዕሚ ኣይስማዕማዕን 2. ኣይስማማዕን<br>3. ሓሳብ የብለይን 4. ተሰማሚዐ 5. ብጣዕሚ ይስማዕማዕ
4.10.	እንድሕር ናብ ኣፍ ማህፀን ካንሰር ሃሊውኺ ብዘይምፍራሕ ንዶክተር ተማክሪ ዶ?	1. ብጣዕሚ ኣይስማዕማዕን 2. ኣይስማማዕን<br>3. ሓሳብ የብለይን 4. ተሰማሚዐ 5. ብጣዕሚ ይስማዕማዕ

5. ናይ ኣፍ ማህፀን ካንሰር ዘይምምርማር ዝምልከት

**ተ.ቁ**	**ሕቶታት**	**መልሲ**
5.1.	ቅድሚ ሐዚ ናይ ኣፍ ማህፀን ካንሰር ዘይምፍታሽ/ዘይምርማር ምክንያትኪ እንታይ እዩ?	1. ግዲ ዘይምህላው 2. ተጋላፂት እየ ዘይምባል<br>3. ጠቃሚ እዩ ኢልካ ዘይምሕሳብ 4. ምስ ስብኣይካ ብሓሳብ ዘይምስምዕማዕ<br>5. ክባር ዋጋ ምርመራ 6. ናይ ካንሰር ምርመራ ውፂኢት ምፍራሕ<br>7. ናይ ማህፀን ካንሰር መመርመሪ መሳርሒ ቃንዛ ምፍራሕ<br>8. ናይ ማህፀን ካንሰር መመርመሪ መሳርሒ ኣበይ ከም ዝርከብ ዘይምፍላጥ<br>9. ናይ ማህፀን ካንሰር መመርመሪ ቦታ ኣብ ቀረባ ዘይምህላው<br>10. እምነት ኣብ ሃይማኖት ምህላው 11. ናይ ሓኪም ምክሪ ምስኣን<br>12. ካልእ_______________________
5.2.	ኣብ ናይ ኣፍ ማህፀን ካንሰር ምርመራ ምስ ስብኣይኪ ምኽሪ ትገብሪ ዶ?	1. እወ 2. ኣይፈልጥን

6. **ርሕቀት ናይ ኣፍ ማህፀን ካንሰር ምርመራ ቦታ ዝምልከት**

**ተ.ቁ**	**ሕቶታት**	**መልሲ**
6.1.	ናይ ኣፍ ማህፀን ካንሰር ምርመራ ቦታ ሰሚዕኪ ትፈልጢ ዶ?	1. እወ 2. ኣይፈልጥን
6.2.	ናይ ኣፍ ማህፀን ካንሰር ምርመራ ቦታ ካብ ገዛኺ ክንደይ ይርሕቅ?	_____ኪ/ሜትር 1. ብጣዕሚ ቀረባ 2. ቀረባ<br>3. ማእኸላይ 4. ርሑቅ 5. ብጣዕሚ ርሑቅ
6.3.	ናብ ኣፍ ማህፀን ካንሰር ምርመራ ቦታ መጓዐዓዚ እንታይ እዩ?	1. ናይ እግሪ ጉዕዞ 2. ብናይ ህዝቢ መጓዐዓዚ መኪና<br>3. ካሊእ____________________

6.4.	እንድሕር ብናይ ህዝቢ መጓዐዓዚ መኪና ኮይኑ ክንደይ ቕርሺ ትከፍላ?	_______________
6.5.	ናይ መኪና መጓዐዓዚ ዋጋ ከመይ ትርኢኦ ?	1. ማእከላይ 2. ክባር

ን ተሳተፎኹን ካብ ልቢ ነመስግን !
